# First incidence study of the site surgical infection in the obstetrics and gynecology services during the years 2008-2010 in Bologhine Hospital.DZ

**DOI:** 10.1186/1753-6561-5-S6-P187

**Published:** 2011-06-29

**Authors:** W Amhis, H Tirchi, K Hadjar

**Affiliations:** 1 Laboratoire Central de Biologie, Hôpital Bologhine Ibn Ziri Algiers, Algiers, Algeria

## Introduction / objectives

The aim of this study is to evaluate the incidence of the site surgery infection (SSI) in the obstetrics and the gynecology services in our hospital.

## Methods

All patients hospitalised in the two services during this period were included in this study. We used the CDC definitions.

## Results

5797 patients were monitored, including 3530 and 2267 patients of obstetrics and Gynecology. 0.92% and 2.15% of the inpatients in Gynecology and Obstetrics were operated during this period. The infection rate was very high in the obstetrics service (90%) and the SSI represented 94,73%. The infection rate was maybe lower in the Gynecology 35.95% but it was represented essentially by the SSI (80.35%). Patients have undergone an operation during the first day of their admission in 65.27% in obstetrics and the second day in 58.82% in Gynecology. The infection appeared during the first week in 54.16% in obstetrics and the second week in 64.70% in the Gynecology. The surgery was done urgently in 68% for the obstetrics patients and programmed in 88,23% for the gynecology patients. The etiological agents were essentially *S.aureus* and enterobacteria respectively isolated in 27.14% and 25.71% in Obstetrics and 26% for the two genus in Gynecology.

## Conclusion

This first study showed that the SSI rate is very high, the operation is done on an emergency or scheduled. Much work remains to be done in these two services. We have to enhance the hygiene measures around the patient to operate (among others hand hygiene and the use of the alcoholic solution) and maybe involve also surgeons in transmitting their own infection rates.

## Disclosure of interest

None declared.

